# Metastatic Malignant Melanoma Mimicking Benign Breast Cysts

**DOI:** 10.1155/2011/245832

**Published:** 2011-07-09

**Authors:** Marius Lund-Iversen, Olav Inge Håskjold, Hiep Phuc Dong, Aasmund Berner

**Affiliations:** ^1^Department of Pathology, Oslo University Hospital, Radiumhospitalet, N-0310 Oslo, Norway; ^2^Department of Radiology, Oslo University Hospital, Radiumhospitalet, N-0310 Oslo, Norway; ^3^Faculty of Medicine, University of Oslo, N-0310 Oslo, Norway

## Abstract

Benign cysts are one of the most common mass-occupying lesions of the breast and are often investigated with triple diagnostic trial (clinical examination, radiology, and cytology). Malignant melanoma is one of medicine's imitators, and metastatic disease can mimic cysts. Thorough investigation of any breast mass is essential to clarify its nature.

## 1. Case Report

**Figure 1:**
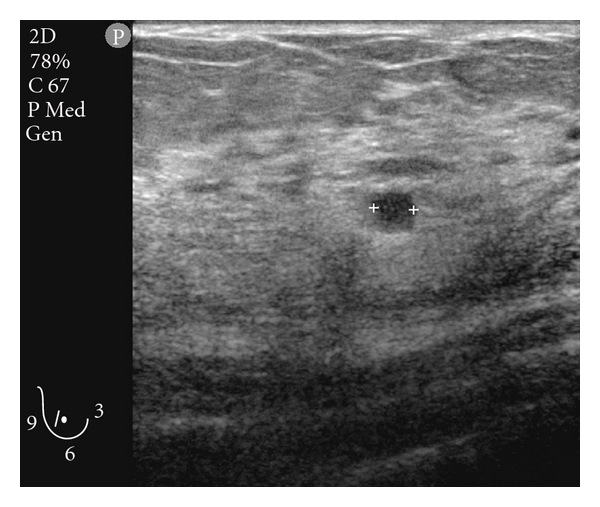
Hypoechoic lesion with demarcated margins and weak echo enhancement.

**Figure 2:**
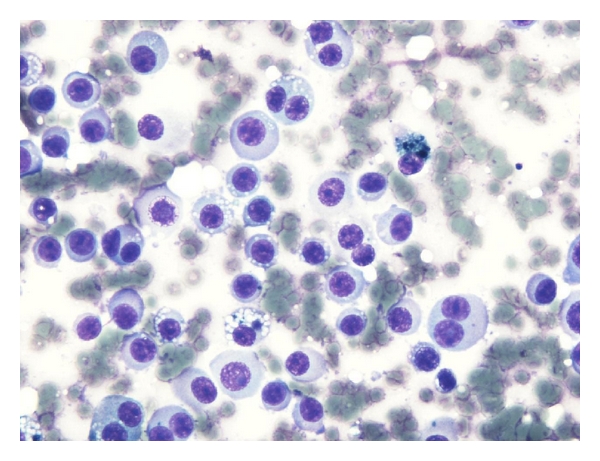
Smear dominated by large mononuclear cells with slightly condensed basophilic cytoplasm and enlarged nucleoli.

**Figure 3:**
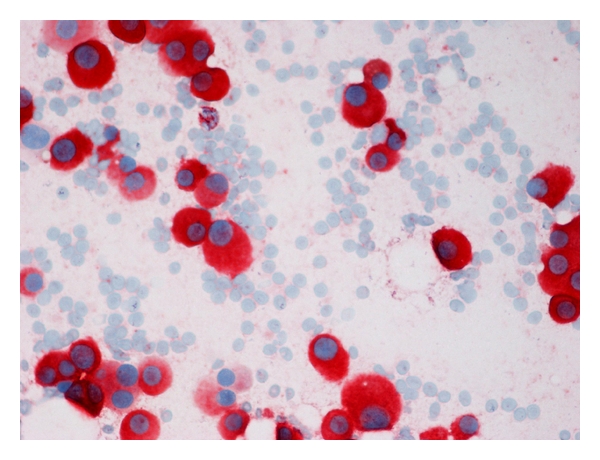
Mononuclear cells positive for Melan-A, consistent with metastatic cells.

A 42-year-old woman presented with bilateral lesions of the breasts which on ultrasound examination were considered hypoechoic cyst-like lesions with sharply demarcated margins (Figure 1). FNAC (fine needle aspiration cytology) was performed as part of the triple test, and air-dried, May-Grünwald-Giemsa-stained smears interpreted by the cytopathologist on duty revealed dissociated mononuclear cells of uncertain origin (Figure 2). Ductal and apocrine epithelia were absent. Scattered cells had uncharacteristic pigment with slightly enlarged nuclei. The cytopathologist ordered a repeat FNAC for ancillary tests. Flow cytometry was inconclusive without distinct populations, probably due to low cellularity and fragile cells which were destroyed during the staining procedure. Immunocytochemistry was positive for Melan-A (Figure 3), negative for cytokeratin. Final diagnosis was consistent with malignant melanoma, probably metastases. The patient died 10 months later due to widespread disease.

## 2. Discussion

FNAC is commonly used as part of the diagnostic triad, which in addition includes clinical breast examination and ultrasonography. The diagnostic accuracy is close to 100% when all three modalities favour a benign or malignant diagnosis [1]. Metastases are rarely found in the breast. Vergier et al. published that approximately 2% of breast tumours are metastases [2]. In our hospital malignant melanoma is the most common primary cancer for metastatic disease to the breast [3]. Specific ultrasound description of metastatic malignant melanoma in the breast is sparse, but the “cyst-like” hypoechogenic character with sharply demarcated margins has previously been reported [4, 5].

This case demonstrates that not all cyst-like breast lesions are benign, and morphologic examination of cyst content by experienced cytopathologist may contribute to final diagnosis or change of further diagnostic approach. 

Unknown to the investigators she had, five months prior to the breast investigation, underwent a neck dissection with superficial parotidectomy, and histological examination of the specimen showed metastasis from malignant melanoma. In search for primary focus, skin examination by dermatologist was negative, but a PET scan showed unspecific uptake in one breast, and she was admitted to the breast centre.

Median survival after diagnosis of breast metastases from malignant melanoma is 12,9 months [4]. Surgery is the choice of treatment in absence of other metastases [6]. Malignant melanoma metastasis in the breast usually occurs in advanced-stage disease and the benefit of early detection is not known.

For adequate treatment, independent of prognosis, any malignancy should be classified to the correct stadium. It is therefore essential for the radiologist and pathologist to be aware of the pitfalls in diagnostics.
